# Pharmacokinetic Estimation Models-based Approach to Predict Clinical Implications for CYP Induction by Calcitriol in Human Cryopreserved Hepatocytes and HepaRG Cells

**DOI:** 10.3390/pharmaceutics13020181

**Published:** 2021-01-29

**Authors:** Yoon-Jee Chae, Min-Soo Kim, Suk-Jae Chung, Mi-Kyung Lee, Kyeong-Ryoon Lee, Han-Joo Maeng

**Affiliations:** 1 College of Pharmacy, Woosuk University, Wanju-gun 55338, Korea; yjchae@woosuk.ac.kr (Y.-J.C.); leemk@woosuk.ac.kr (M.-K.L.); 2 College of Pharmacy and Research Institute of Pharmaceutical Sciences, Seoul National University, Seoul 08826, Korea; misol@snu.ac.kr (M.-S.K.); sukjae@snu.ac.kr (S.-J.C.); 3 Laboratory Animal Resource Center, Korea Research Institute of Bioscience and Biotechnology, Cheongju 28116, Korea; 4 College of Pharmacy, Gachon University, Incheon 21936, Korea

**Keywords:** calcitriol, CYP induction, human cryopreserved hepatocytes, HepaRG, basic model, static mechanistic model, PBPK model

## Abstract

Calcitriol, a vitamin D_3_ metabolite, is approved for various indications because it is the bioactive form of vitamin D in the body. The purpose of this study was to predict the clinical significance of cytochrome P450 (CYP) induction by calcitriol using in vitro human cryopreserved hepatocytes, HepaRG experimental systems, and various pharmacokinetic estimation models. CYP2B6, 3A4, 2C8, and 2C9 mRNA levels increased in a concentration-dependent manner in the presence of calcitriol in human cryopreserved hepatocytes and HepaRG cells. Using the half maximal effective concentration (EC_50_) and maximum induction effect (E_max_) obtained from the in vitro study, a basic kinetic model was applied, suggesting clinical relevance. In addition, a static mechanistic model showed the improbability of a clinically significant effect; however, the calculated area under the plasma concentration–time curve ratio (AUCR) was marginal for CYP3A4 in HepaRG cells. To clarify the effect of CYP3A4 in vivo, physiologically based pharmacokinetic (PBPK) modeling was applied as a dynamic mechanistic model, revealing a low clinically significant effect of CYP3A4 induction by calcitriol. Therefore, we conclude that CYP induction by calcitriol treatment would not be clinically significant under typical clinical conditions.

## 1. Introduction

Vitamin D_3_ is produced when the skin is exposed to sunlight and can be acquired in the diet from animal sources such as fish oil and egg yolks. Vitamin D_3_ is metabolized to calcidiol (25-hydroxyvitamin D_3_; 25(OH)D_3_) by vitamin D_3_-25-hydroxylase in the liver. Subsequently, it is metabolized by 25-hydroxylase in the renal tubules to produce calcitriol (1,25-dihydroxyvitamin D_3_; 1,25(OH)_2_D_3_), which is an active form of vitamin D [1,2]. The main function of calcitriol is the regulation of calcium and phosphate homeostasis by increasing the absorption of calcium and phosphate in the gastrointestinal tract and decreasing parathyroid hormone synthesis [1,3].

As calcitriol is the bioactive form of vitamin D in the body, calcitriol itself is approved for use in patients with certain diseases. For example, Rocaltrol^®^ is a representative oral formulation of calcitriol developed by Roche and is orally available as either a capsule (0.25 μg or 0.5 μg) or an oral solution (1 μg/mL). It is indicated in the management of secondary hyperparathyroidism and resultant metabolic bone disease in patients with moderate to severe chronic renal failure who are not yet on dialysis; hypocalcemia and resultant metabolic bone disease in patients undergoing chronic renal dialysis; and hypocalcemia and its clinical manifestations in patients with postsurgical hypoparathyroidism, idiopathic hypoparathyroidism, and pseudohypoparathyroidism [4]. Calcitriol is also available as intravenous solutions such as Calcijex^®^ (Abbott Laboratories), which is indicated in the management of hypocalcemia in patients undergoing chronic renal dialysis [5].

In addition to the approved indications described above, the use of calcitriol in other conditions continues to be suggested. Several preclinical studies have provided considerable evidence for the antitumor effect of calcitriol using various in vitro/in vivo experimental systems [6,7,8,9,10,11]. In addition, clinical studies have suggested a promising antitumor effect of calcitriol [12,13,14] although further investigation and confirmation are needed. Besides the antitumor effect, recent studies have reported that calcitriol is associated with cell proliferation and differentiation as well as immune and inflammatory responses [7,15,16,17,18,19,20,21,22,23]. In many of these studies, calcitriol was used as a combination therapy to increase the therapeutic efficacy of drugs that are already in clinical use. When employing a combination regimen, information on drug–drug interactions (DDIs) regarding pharmacokinetics as well as pharmacological pathways is necessary to facilitate more efficient and safe therapies. However, most studies on calcitriol combination strategies have focused only on pharmacological interactions. Considering that dramatic pharmacokinetic DDIs have been reported in several combination treatments in humans [24,25,26,27,28], more detailed and concrete information on the pharmacokinetic interactions of calcitriol is necessary to evaluate the clinical significance of DDIs.

Cytochrome P450s (CYPs) are the most important family of enzymes affecting pharmacokinetic profiles of not only endogenous substrates but also many drugs. CYPs are responsible for approximately 75% of the metabolism of commercially available drugs [29]. Thus, DDIs via CYPs need to be emphasized when investigating the clinical implications of pharmacokinetic interactions. 

It has been reported that CYPs such as CYP3A4, 2B6, 2C8, and 2C9 are inducible by calcitriol treatment in vitro [30,31]. However, there is a lack of information regarding the clinical significance of CYP induction by calcitriol. If this induction were clinically significant, we would expect that the pharmacokinetic profiles of co-administered drugs that are metabolized by the induced CYPs would be altered, consequently changing their pharmacodynamics or toxicological patterns. Thus, it is crucial to predict the impact of CYP induction by calcitriol in clinical use to provide more effective and safe treatment regimens.

The United States Food and Drug Administration (FDA) released guidelines in 2020 regarding in vitro studies to estimate the clinical significance of DDIs [32]. This guideline contains a detailed strategy to evaluate the potential for pharmacokinetic interactions. Briefly, summarizing the strategy for investigating the induction of drug-metabolizing enzymes, the guidance suggests that CYPs such as CYP1A2, 2B6, 2C8, 2C9, 2C19, and 3A4 be evaluated for DDI potential using a step-wise approach (i.e., from a basic model to a mechanistic model). In addition, these guidelines suggest using human hepatocytes or alternative in vitro systems such as immortalized hepatic cell lines. HepaRG cells are widely used in vitro to mimic human liver and their drug metabolism profiles are comparable to those of human hepatocytes [33,34]. If an in vitro assessment performed according to these guidelines suggests clinically significant DDIs, a clinical DDI study should then be conducted [35]. This strategy is a systematic and risk-based approach and is considered the gold standard approach to assess DDI potential [36]. Therefore, we aimed to estimate the clinical significance of CYP induction by calcitriol using this step-wise approach in the present study. To predict the clinical implications, we performed a CYP induction study in vitro and utilized various pharmacokinetic estimation models, such as the basic kinetic model and mechanistic model.

## 2. Materials and Methods 

### 2.1. Materials

Cryopreserved human hepatocytes from three donors (#1: HMC 514, #2: HFC920, #3: HMC1034), the High Viability Cryohepatocyte Recovery Kit, Hepatocyte Culture Media Kit, Matrigel matrix, and collagen I-coated 24-well plates were obtained from Corning Life Sciences (Woburn, MA, USA). GlutaMAX-I supplement was purchased from Gibco (Grand Island, NY, USA). Cryopreserved HepaRG cells, HepaRG Thaw, Plate, and General Purpose Medium Supplement, and HepaRG Induction Medium Supplement were obtained from Biopredic (Rennes, France). Calcitriol, omeprazole, 6-(4-chlorophenyl)imidazo[2,1-b] [1,3]thiazole-5-carbaldehyde O-3,4-dichlorobenzyl) oxime (CITCO), rifampin, bupropion, and William’s Medium E were purchased from Sigma-Aldrich (St. Louis, MO, USA). Superscript III First-Strand Synthesis SuperMix, TRIzol, and diethyl pyrocarbonate (DEPC)-treated water were obtained from Invitrogen (Carlsbad, CA, USA). Quantitative polymerase chain reaction (qPCR) reagents were purchased from Applied Biosystems (Foster City, CA, USA). Dimethyl sulfoxide (DMSO) was obtained from Merck (Merck-Millipore, Darmstadt, Germany). All other chemicals were of reagent grade or better and were used without further purification.

### 2.2. HepaRG Seeding and Culture

On the day of seeding, the thawing and plating medium was prepared by adding the HepaRG Thaw, Plate, and General Purpose Medium Supplement and GlutaMAX-I into William’s Medium E. Cryopreserved HepaRG cells were thawed in a 37 °C water bath and transferred into a tube containing the thawing and plating medium. After centrifugation, the supernatant was aspirated carefully, and thawing and plating medium was added. The cells were resuspended by pipetting and the number of viable cells was counted with a hemocytometer after trypan blue staining. The HepaRG cells were diluted to 1 × 10^6^ cells/mL in the thawing and plating medium and 400 μL of the cell suspension was transferred per well to collagen I-coated 24-well plates. The plate was gently tapped and placed in a 37-°C CO_2_ incubator. After seeding for approximately 4 h, the medium was replaced with fresh thawing and plating medium, and the cells were further incubated at 37 °C in the CO_2_ incubator.

### 2.3. Human Hepatocyte Seeding and Culture

Cryopreserved hepatocytes were thawed in a 37-°C water bath and transferred into a recovery medium tube from the High Viability Cryohepatocyte Recovery Kit. After centrifugation at 100 g for 10 min, the supernatant was aspirated carefully and prewarmed plating medium was added. The cells were resuspended by pipetting and the number of viable cells was counted with a hemocytometer after trypan blue staining. The hepatocytes were diluted to 1 × 10^6^ cells/mL using plating medium and 400 μL of the cell suspension was transferred per well to collagen I-coated 24-well plates. The plate was placed in a 37 °C CO_2_ incubator and tapped gently every 20–30 min for 2 h to distribute the cells evenly. After seeding for approximately 4 h, the medium was replaced with 500 μL of Hepatocyte Culture Media containing 0.25 mg/mL of Matrigel and the cells were further incubated at 37 °C in the CO_2_ incubator.

### 2.4. Test Article Treatment

For the treatment of HepaRG cells with test articles, induction medium was prepared by adding HepaRG Induction Medium Supplement and GlutaMAX-I into William’s Medium E. Seventy-two hours after HepaRG plating, the medium was replaced with freshly prepared induction medium containing the positive control or calcitriol. Twenty-four hours after hepatocyte seeding, the medium was replaced with freshly prepared medium containing the positive control or calcitriol.

50 μM Omeprazole for CYP1A2, 0.1 μM CITCO for CYP2B6, or 10 μM rifampin for CYP3A4/2C8/2C9/2C19 was used as the positive control, respectively. Hepatocytes or HepaRG cells were exposed a range of calcitriol concentrations between 1 and 100 nM. The final concentration of the organic solvent carrier was identical in all samples and did not exceed 0.1% (*v*/*v*). After 24 h, the medium was replaced with freshly prepared medium containing the positive control or calcitriol and the incubation was continued for 24 h. All experiments were performed in triplicate.

### 2.5. RNA Isolation and RT-qPCR

Total RNA was isolated using TRIzol reagent according to the manufacturer’s instructions after test article treatment for 48 h. The concentration and purity of the extracted RNA were confirmed by UV spectrophotometry at 260/280 nm. Reverse transcription (RT) was then performed using Superscript III First-Strand Synthesis SuperMix. For qPCR, the TaqMan^®^ Gene Expression Master Mix and TaqMan^®^ probe specific for each gene (*glyceraldehyde-3-phosphate dehydrogenase* (*GAPDH*): Hs02758991_g1, *CYP1A2*: Hs01070369-m1, *CYP2B6*: Hs03044634-m1, *CYP3A4*: Hs00430021_m1, *CYP2C8*: Hs00426387_m1, *CYP2C9*: Hs00426397_m1, and *CYP2C19*: Hs00426380_m1) were used. The thermal cycler protocols were as follows: enzyme activation/initial denaturation at 95 °C for 10 min, followed by 40 cycles of denaturation at 95 °C for 15 s and annealing/extension at 60 °C for 1 min. mRNA fold-induction was calculated using the 2^−ΔΔCT^ method.

### 2.6. CYP Activity Test

After the test article treatment for 48 h, the cells were washed twice with William’s Medium E and 200 μL of this medium containing 100 μM bupropion (CYP2B6 substrate) and 50 μM testosterone (CYP3A4 substrate) was added to each well. The cells were incubated for 30 min at 37 °C in a CO_2_ incubator. After the incubation, 80 μL of each sample was transferred into a microcentrifuge tube, 80 μL of acetonitrile containing the internal standard (i.e., verapamil) was added, and the mixture was centrifuged at 12,000 rpm for 5 min. The supernatant was then used for liquid chromatography-tandem mass spectrometry (LC-MS/MS) analysis. Hydroxybupropion and 6β-hydroxytestosterone were analyzed as CYP2B6 and CYP3A4 metabolites, respectively.

The LC-MS/MS system was comprised of an Agilent 1200 series HPLC and 6460 triple quadrupole mass spectrometer. Chromatographic separation was performed using a Zorbax SB-C18 column (2.1 × 30 mm, 3.5 μm, Agilent, Santa Clara, CA, USA) under gradient conditions using mobile phase A (0.1% formic acid in water) and B (0.1% formic acid in acetonitrile). Analyte detection was performed using multiple reaction monitoring (MRM) transitions in electrospray positive ionization (ESI+) mode. The transition of precursor ion to product ion was m/z 256.1 to 238.1 for hydroxybupropion and m/z 305.3 to 269.1 for 6β-hydroxytestosterone.

The peak area ratio in the MS chromatogram was calculated by dividing the peak area of the analyte by the peak area of the internal standard. Metabolite formation was expressed as fold induction over the control group.

### 2.7. Determination of E_max_ and EC_50_

*E_max_*, the maximum induction effect, and *EC_50_*, the half maximal effective concentration, were determined using the mRNA fold-induction observed in hepatocytes and HepaRG cells. The sigmoidal dose–response function was utilized in GraphPad Prism 8.4.2 (GraphPad Software, San Diego, CA, USA).

### 2.8. Prediction of Clinical Significance of CYP Induction by Calcitriol—Basic Kinetic Model

Based on the FDA guidance on in vitro DDI studies [32], a basic kinetic model was applied as a first step; the equation used is shown below (1).

(1) $$R_{3}=\frac{1}{1+d\times (\frac{E_{max}\times 10\times I_{max,u}}{EC_{50}+10\times I_{max,\;u}})}$$

Here, *R_3_* is the predicted ratio of intrinsic clearance values of a probe substrate for an enzymatic pathway in the absence and presence of an inducer, d is the scaling factor, *E_max_* is the maximum induction effect determined in vitro, and *I_max,u_* is the maximal unbound plasma concentration of the interacting drug at steady state. To calculate *I_max,u_*, three maximum plasma concentrations (*C_max_*) from various clinical conditions were considered: (1) calcitriol standard oral regimen: steady state *C_max_* after intake of 1 μg calcitriol (assumed maximum dose of oral administration for the approved indication) was used [37]; (2) calcitriol standard intravenous regimen: *C_max_* after intravenous administration of 4 μg calcitriol (assumed maximum dose of intravenous injection for the approved indication) [38]; and (3) calcitriol high-dose intravenous regimen: *C_max_* after intravenous administration of 74 μg calcitriol (the reported maximum tolerable dose (MTD) of calcitriol with intravenous injection) [39]. The unbound fraction of calcitriol was assumed to be 1% because it has been reported that 99.9% of calcitriol exists in a bound form [37] and it is reasonable to set the unbound fraction to 1% in this case according to FDA guidelines. The predefined *R_3_* cut-off value to determine DDI potential was set to 0.8 (*R_3_* ≤ 0.8 means that calcitriol has induction potential in vivo).

### 2.9. Prediction of Clinical Significance of CYP Induction by Calcitriol—Static Mechanistic Model

Further investigations on the clinical implication of CYP induction by calcitriol were performed using a static mechanistic model. Equation (2) was used to calculate the area under the plasma concentration–time curve ratio (*AUCR*), which indicates the overall effect of calcitriol on substrate drugs.

(2) $$\mathrm{AUCR}=(\frac{1}{\left[A_{g}\times B_{g}\times C_{g}\right]\times \left(1-F_{g}\right)+F_{g}})\times (\frac{1}{\left[A_{h}\times B_{h}\times C_{h}\right]\times f_{m}+\left(1-f_{m}\right)})$$

Here, *A*, *B*, and *C* are the effects of reversible inhibition, time-dependent inhibition, and induction, respectively. *A* and *B* are assumed to be 1 according to the United States Food and Drug Administration (US FDA) guidelines (i.e., when mechanistic models are used for predicting DDIs caused by enzyme induction, the model should include induction mechanisms only) [32].

The definitions, equations, and values of each parameter used to calculate the *AUCR* are presented in Table 1. When source data were not available from references, the most conservative assumption was used. If the calculated *AUCR* was >0.8, we concluded that there was no clinically significant impact of DDIs via CYP induction based on FDA guidance [32].

### 2.10. Prediction of Clinical Significance of CYP Induction by Calcitriol—Dynamic Mechanistic Model (PBPK Model)

A physiologically based pharmacokinetic (PBPK) model was utilized as a dynamic mechanistic model to predict the clinical significance of CYP3A4 induction by intravenous administration of 74 μg calcitriol. The PBPK model was developed using Simcyp Simulator version 19 (Certara UK Limited, Sheffield, United Kingdom). The plasma concentration data of calcitriol after intravenous administration of 74 μg calcitriol were extracted from a previously reported study [39]. The dosing schedule was set to days 1, 15, 22, and 29, which matches the schedule performed in the clinical study with the cancer patients. Midazolam was chosen as a probe substrate for CYP3A4, and the pharmacokinetic profiles of midazolam after oral administration of 3.75 mg midazolam in cancer patients were simulated using the validated PBPK models from the Simcyp library (i.e., Sim-Midazolam and Sim-Cancer) [44,45,46]. The full PBPK model approach was applied to calcitriol and the *K_p_* values were estimated using the Rodgers and Rowland method [47]. Simulations were performed with a total of 200 cancer patients grouped into 10 trials. The virtual subjects were between 20 and 60 years old and the ratio of males to females in the population was 1:1. Detailed information on the input parameters for the PBPK modeling is shown in Table S1 in the Supplementary Materials.

### 2.11. Data Analysis

Data are expressed as the means ± standard deviations. Student’s *t*-tests were used for statistical comparisons.

## 3. Results

3.1. mRNA Induction Study

**Figure 1:**
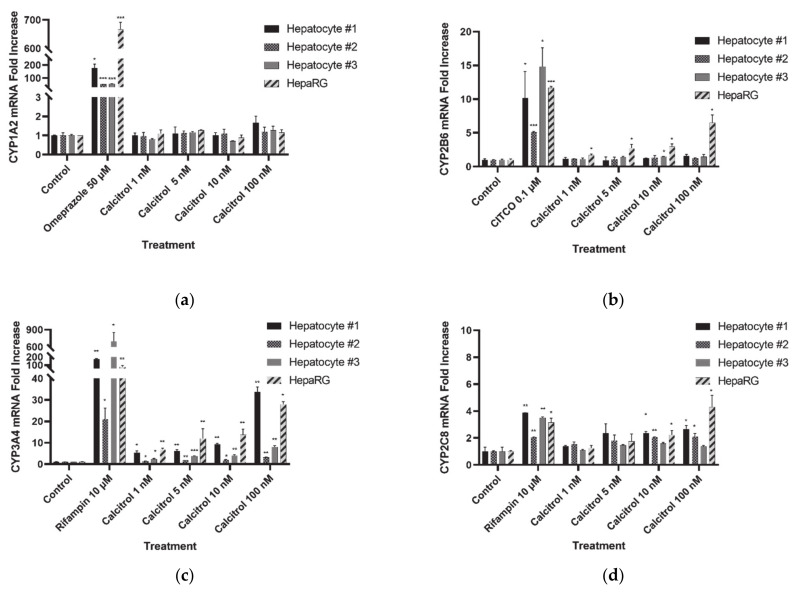

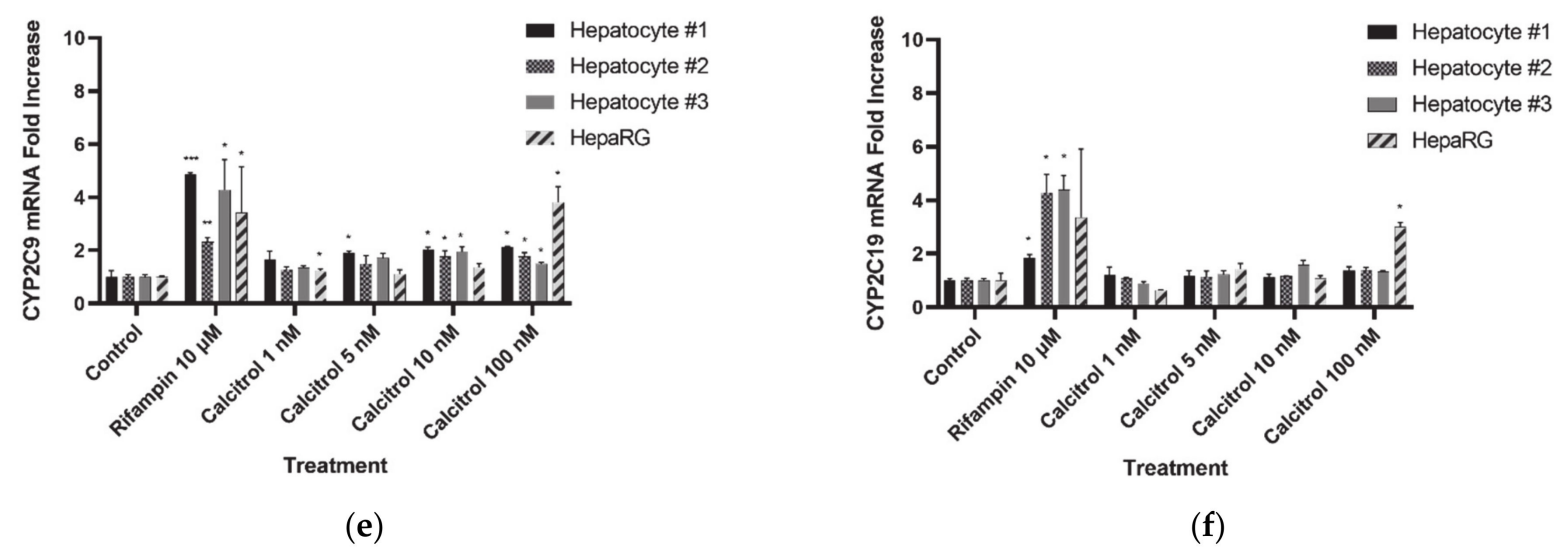
mRNA expression level of cytochrome P450s (CYPs) after treatment with positive control or calcitriol in human cryopreserved hepatocytes and HepaRG cells. (**a**) *CYP1A2*; (**b**) *CYP2B6*; (**c**) *CYP3A4*; (**d**) *CYP2C8*; (**e**) *CYP2C9*; and (**f**) *CYP2C19*. Data are shown as the means ± standard deviations. * *p* < 0.05, ** *p* < 0.01, and *** *p* < 0.001.

We evaluated the ability of calcitriol to induce the CYPs recommended by the FDA guidelines, specifically CYP1A2, 2B6, 3A4, 2C8, 2C9, and 2C19, which are recognized as the most prominent CYPs for drug metabolism [32,48,49]. We used human cryopreserved hepatocytes and HepaRG cells, which are widely used to investigate drug metabolism. Each positive control (omeprazole for CYP1A2, CITCO for *CYP2B6* and rifampin for *CYP3A4*, *2C8*, *2C9*, and *2C19*) was able to induce mRNA expression levels significantly, indicating that the experimental system and conditions were reliable for this study (Figure 1). Treatment with various concentrations of calcitriol did not induce a significant change in *CYP1A2* mRNA expression levels (Figure 1a). *CYP2B6* mRNA expression levels increased significantly after treatment with 10 nM calcitriol in hepatocytes from donor #2, but the induction was less than two-fold (1.49 ± 0.02) and was not concentration-dependent. This induction was not observed in the hepatocytes from the other donors (#1 and #3). However, *CYP2B6* mRNA expression levels increased in a concentration-dependent manner in HepaRG cells showing statistically significant differences compared to the control (fold increase: 1.70 ± 0.15, 2.76 ± 0.56, 2.96 ± 0.39, and 6.51 ± 1.14 (all *p* < 0.05) after treatment with 1, 5, 10, and 100 nM calcitriol, respectively) (Figure 1b). *CYP3A4* mRNA expression levels increased in a concentration-dependent manner, with a statistically significant difference in the hepatocytes originating from the three donors and HepaRG cells, although the absolute fold-increases were different among the hepatocytes (Figure 1c). Statistically significant induction of *CYP2C8* mRNA was observed only after treatment with a high concentration of calcitriol (10 or 100 nM) and the fold-induction was ≤3 in human hepatocytes, while it was 4.31-fold after treatment with 100 nM calcitriol in HepaRG cells (Figure 1d). *CYP2C9* mRNA was induced approximately 2-fold by treatment with the highest concentration of calcitriol (100 nM) in human hepatocytes; however, the induction was 3.82-fold with the same treatment in HepaRG cells (Figure 1e). *CYP2C19* mRNA expression levels were not significantly increased in human hepatocytes, whereas they were upregulated 3.00-fold following treatment with 100 nM calcitriol in HepaRG cells (*p* < 0.05, Figure 1f). Although the extent of induction effect or profiles of mRNA expression levels by calcitriol treatment were observed to be somewhat different between hepatocytes and HepaRG cells, overall trends in mRNA induction appeared consistent between the two systems.

### 3.2. CYP Activity Test

**Figure 2:**
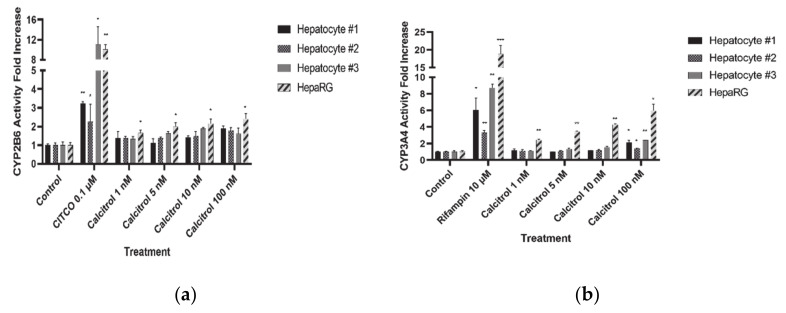
CYP activity measurement after treatment with positive control or calcitriol in human cryopreserved hepatocytes and HepaRG cells. (**a**) CYP2B6 and (**b**) CYP3A4. Data are shown as the means ± standard deviations. * *p* < 0.05, ** *p* < 0.01, and *** *p* < 0.001.

Metabolic activity was measured for CYP2B6 and CYP3A4, which had shown significant and concentration-dependent increases in mRNA levels. Consistent with the qPCR results, a statistically significant increase in CYP2B6 activity was observed in the HepaRG cells, but not in the hepatocytes (Figure 2a). In the case of CYP3A4, its activity increased in a concentration-dependent manner in HepaRG cells, whereas it increased significantly only with the highest concentration of calcitriol (100 nM) in human hepatocytes (Figure 2b). The magnitude of the increased activity was much less than that of the increased mRNA expression.

### 3.3. Determination of E_max_ and EC_50_

**Figure 3:**
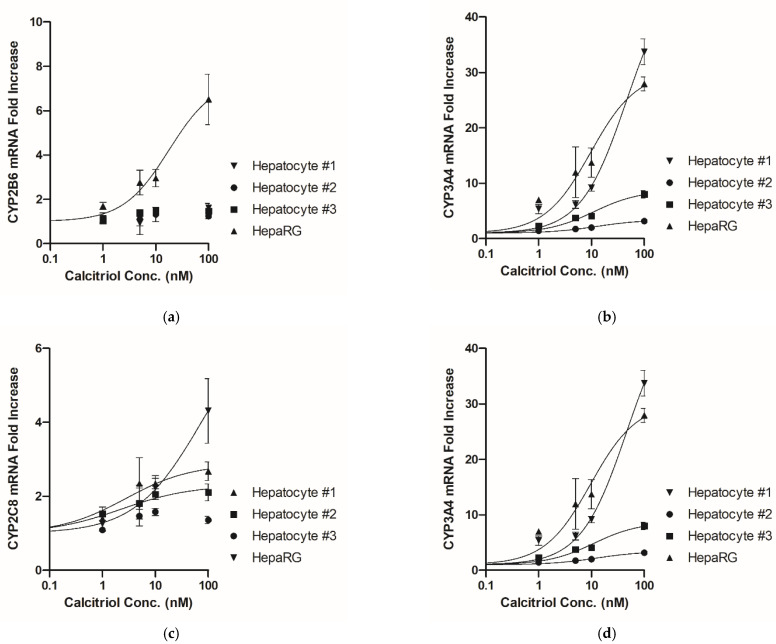
Concentration–response curve for CYP mRNA induction by treatment with calcitriol in human cryopreserved hepatocytes and HepaRG cells. A sigmoidal dose-response function was utilized. (**a**) *CYP2B6*; (**b**) *CYP3A4*; (**c**) *CYP2C8*; and (**d**) *CYP2C9*. Data are shown as the means ± standard deviations.

*E_max_* and *EC_50_* were estimated using mRNA fold-induction by applying a sigmoidal dose–response model (Figure 3). The magnitude of the induction profiles for CYP activity was much less than that for mRNA; thus, only the mRNA induction results were used. The *E_max_* and *EC_50_* for *CYP3A4* were calculated in the hepatocytes from all three donors as well as the HepaRG cells; however, these parameters were not available for *CYP2B6*, *CYP2C8*, and *CYP2C9* in the hepatocytes from certain donors. *CYP1A2* and *CYP2C19* were excluded from the calculation of these parameters since their mRNA levels were not increased or the increase was not concentration-dependent. The estimated *E_max_* and *EC_50_* values are presented in Table 2.

### 3.4. Prediction of Clinical Significance of CYP Induction by Calcitriol Using a Basic Kinetic Model

Using the *E_max_* and *EC_50_* values and equation (1), R_3_ values were calculated and are shown in Table 3. When the clinical situation of oral administration of 1 μg calcitriol was assumed, which is the currently approved maximum oral dose, the calculated *R_3_* value for CYP2B6 was higher than 0.8, the predefined cut-off value. The R_3_ values for CYP3A4, 2C8, and 2C9 with oral administration of 1 μg calcitriol were less than 0.8 in hepatocytes and HepaRG cells, indicating that the clinical implications of these isoforms should be further investigated with other models, such as the static mechanistic model. The prediction with the calcitriol standard or high-dose intravenous regimen also highlighted the necessity of investigating clinical implications further because the calculated *R_3_* values were less than 0.8 in most cases for CYP2B6, 3A4, 2C8, and 2C9.

### 3.5. Prediction of Clinical Significance of CYP Induction by Calcitriol Using Static Mechanistic Model

Since the results from the basic kinetic model could not exclude the potential of clinically significant DDIs via CYP induction, further investigation of the CYP induction potential was performed using a static mechanistic model. Regardless of the CYP isoform and assumed dose regimen, the calculated AUCR was more than 0.8, which is the predefined cut-off value for the determination of clinical significance (Table 4). However, the calculated *AUCR* for CYP3A4 in HepaRG cells with the high-dose intravenous regimen was 0.802, which is very close to the predefined cut-off value (0.8).

### 3.6. Prediction of Clinical Significance of CYP3A4 Induction by Calcitriol Using PBPK Model

**Figure 4:**
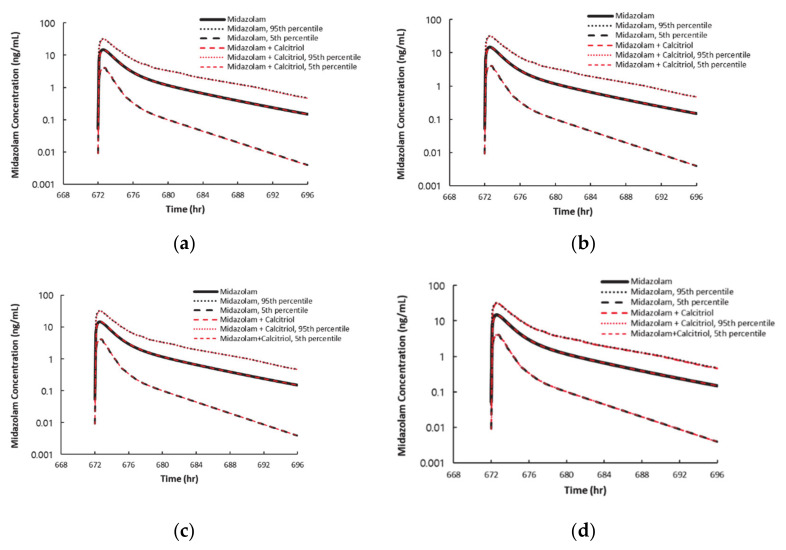
Predicted concentration–time profiles of midazolam. *EC_50_* and *E_max_* originating from Hepatocytes #1 (**a**), #2 (**b**), #3 (**c**), and HepaRG cells (**d**) were used. The black line represents predicted concentration–time profiles of midazolam after 3.75 mg oral administration without calcitriol treatment. The red dashed line represents predicted concentration–time profiles of midazolam on day 29 after 3.75 mg oral calcitrol administration on days 1, 14, 15, and 29.

To further clarify the clinical implication of CYP3A4 induction by calcitriol, PBPK modeling was applied as a dynamic mechanistic model. The simulated calcitriol concentration profiles fitted well to the observed profiles after intravenous administration of 74 μg calcitriol (Figure S1 in Supplementary Materials). Using the simulated calcitriol data, the clinical implication of CYP3A4 induction by calcitriol was predicted. The calculated ratio of the PK parameters on day 29 (PK parameters of midazolam divided by the PK parameters of midazolam when coadministered with 74 μg calcitriol on day 29) was 0.9969–0.9998 when using the *EC_50_* and *E_max_* calculated in hepatocytes and HepaRG cells, indicating that 74 μg calcitriol does not affect the pharmacokinetics of midazolam. When sensitivity analysis was performed using various *E_max_* or *EC_50_* values (e.g., 100-fold higher *E_max_* or 100-fold lower *EC_50_*), the calculated ratio was similar to 1.00, suggesting that the significance of CYP3A4 induction in clinical settings is likely negligible (data not shown). The PK parameters and concentration–time profiles of midazolam estimated using the PBPK model with or without calcitriol treatment are shown in Table 5 and Figure 4.

## 4. Discussion

Information on the pharmacokinetics of DDIs is crucial to ensure effective and safe use of drugs. Lack of information on DDIs may expose patients to increased toxicity risks or decreased efficacy of administered drugs. Regulatory agencies such as the FDA require a substantial amount of information on DDIs for marketing authorization. Therefore, recently approved drugs have usually amassed extensive data to allow safe and effective combination treatment with other drugs in clinical settings. However, there are some cases, especially drugs that have been used for many years, where information regarding DDIs is not sufficient or the interaction potential in clinical settings has not been determined. This may occur because this information was not considered necessary or important at the time of approval.

Calcitriol was approved for medical use in the United States in 1978. It is known to induce CYPs based on several in vitro and in vivo preclinical studies [30,31,50]; however, the clinical implications of its DDIs have not been reported to date. In the present study, we aimed to predict the clinical significance of such DDIs using in vitro hepatocyte and HepaRG experiment systems and various pharmacokinetic estimation models. Calcitriol is a metabolite of vitamin D_3_ which is commonly used as a nutrient supplement. In addition, calcitriol itself is marketed in oral or intravenous formulations because it is a bioactive form of vitamin D in the human body. Considering that the systemic exposure to calcitriol is much higher when exogenously administered than when generated from vitamin D_3_ metabolism within the body, the former scenario was only considered in this study.

The induction effect of drugs can be investigated by measuring mRNA expression levels or functional activity [32]. Although the increase in enzyme activity is significantly correlated with mRNA upregulation, the increase in CYP activity is usually much less than the mRNA increase, suggesting that mRNA levels are more sensitive markers than enzymatic activity [51,52]. In the present study, activities were measured for CYPs with mRNA upregulations that reached a certain level (i.e., CYP2B6 and CYP3A4); the increase in CYP activity was much less than the magnitude of mRNA induction for both of them, as expected. Therefore, further investigation to predict the clinical significance of calcitriol was undertaken using only mRNA expression levels to allow more sensitive and conservative analyses. The different sensitivity of the induction levels between mRNA and activity is often observed when the inducer is also acting as an inhibitor for the metabolic enzyme [51]. Calcitriol, however, is not reported as an inhibitor for CYP2B6 or 3A4 so far. Other plausible explanations might be the involvement of calcitriol in the post-transcriptional regulation of the enzymes [53], or engagement of calcitriol in regulation of other metabolic enzymes that metabolize the probe substrates or their metabolite (i.e., bupropion or hydroxybupropion for CYP2B6, testosterone or 6β-hydroxytestosterone for CYP3A4). However, further study is required to explain the different sensitivity of the induction levels between mRNA and activity and provide more reliable prediction.

To predict the clinical significance of the induction effect of calcitriol, we assumed three scenarios that are most likely to occur in clinical settings. The first scenario was the administration of the maximum approved oral dose of calcitriol (i.e., 1 μg) based on the Rocaltrol^®^ label [4] which showed a *C_max_* of 85 pg/mL [37]. The second scenario was the intravenous treatment regimen (i.e., 4 μg) within the approved dose range of Calcijex^®^ [5], with a *C_max_* of 465 pg/mL at the end of infusion [38]. The third scenario was a combination therapy for the purpose of cancer treatment, with a suggested calcitriol dose of 74 μg, which is the MTD determined in a clinical study. The observed *C_max_* with this third regimen was 6.68 ng/mL [39]. This final scenario is not approved for market yet. However, we decided to include it to assess the most conservative situation; we expect this “worst-case scenario” will ensure that we do not miss any potential DDIs.

The basic kinetic model was used as a first step to predict the clinical implications of CYP induction by calcitriol, and it was applied only to CYPs showing concentration-dependent profiles of mRNA induction with calcitriol. This model uses only simple assumptions about human exposure and does not need to simulate the entire concentration–time profile because a constant value of perpetrator concentration is used to simplify the prediction. Considering this conservative assumption, the basic kinetic model is generally used as a starting point of the prediction. The predefined *R_3_* cut-off value to determine DDI potential was set to 0.8, which is the value described in the FDA guideline on in vitro drug interactions studies [32] as well as bioequivalent study [54] and commonly used in many studies [55,56]. Since most cases showed *R_3_* values less than 0.8, a static mechanistic model was then utilized with a predefined *AUCR* cut-off value of 0.8. The static mechanistic model uses the d-factor to scale the in vitro data to in vivo equivalents and incorporates more detailed drug disposition information. This model can also take into account other concomitant DDIs such as reversible or mechanism-based inhibition; however, only induction-dependent DDIs were assessed in this study to avoid masking the induction effect of calcitriol. Other variables such as *F_g_*, *F_a_*, and *f_m_* were set to the most conservative values to avoid false negative predictions. The clinical effect of CYP induction by calcitriol was estimated not to be significant from the calculated *AUCR* because the values from all cases were numerically higher than 0.8. However, a marginal value was observed for CYP3A4 with the calcitriol high-dose intravenous regimen in HepaRG cells (i.e., *AUCR* = 0.802). Given the acceptable accuracy of quantitative analysis methods or inter-individual pharmacokinetic variability, this value may not guarantee the absence of DDIs in real clinical conditions. Therefore, we proceeded to the next prediction using the PBPK model, which utilizes concentration–time profiles for both perpetrator and victim drugs to predict the clinical significance under more physiological conditions. We used the validated model for midazolam [44,45,46] and developed a PBPK model for calcitriol using the previously reported data [39]. The developed PBPK model was concluded to be valid because the difference of the observed and simulated profiles was minimal as shown in Figure S1 in Supplementary Materials. From this PBPK approach, it was predicted that the pharmacokinetic parameters of midazolam, which is a sensitive probe substrate for CYP3A4, are not altered significantly by treatment with calcitriol. The lack of clinical significance of CYP3A4 induction by calcitriol is believed to be caused by the low systemic exposure of calcitriol not reaching a concentration that is able to influence CYP regulation in the body. In addition, low liver distribution and high fractions of bound calcitriol will result in a small amount of active free calcitriol, which is involved in CYP3A4 induction. The reported *K_p,liver_* value of calcitriol is 0.13 in mice [57] and the estimated *K_p,liver_* value was 0.212 in this study, suggesting that the *K_p,liver_* value used in this study is reasonable. Therefore, these results suggest that the clinical significance of DDIs via calcitriol-depenent CYP induction is likely to be negligible with the calcitriol dose regimen assumed in this study.

Since the small intestine may play an important role in the first-pass metabolism of orally administered drugs [58], the CYPs expressed in the intestine should not be overlooked when predicting the induction effect of CYPs. Moreover, several cases have been reported in which alterations of pharmacokinetics were much greater after oral administration of inducer, which is a perpetrator, compared with intravenous administration and this might be likely due to high concentration of the inducer in the enterocytes when administered orally [59,60,61]. In our study, we considered the induction effect of CYPs in the gut as well as the liver when applying the static mechanistic model as shown in equation (2). While experimental systems to investigate CYP induction in the liver such as cryopreserved hepatocytes or HepRG are well established and widely used, experimental approaches to evaluate the induction effect of CYPs in the gut are still quite limited [62]. As an alternative approach, we used *E_max_* and *EC_50_* values obtained from hepatocytes or HepaRG to calculate *C_g_* (i.e., the effect of induction in gut) to estimate CYP induction effect using static mechanistic model and it concluded that the induction effect of CYPs in the gut was minimal (*C_g_* ranged 1.003–1.03). For more concrete prediction on CYP induction in the gut, *E_max_* and *EC_50_* values for intestinal CYPs need to be obtained from reliable experimental systems in the future.

The antitumor effect of calcitriol has been demonstrated in several in vitro and in vivo preclinical studies [6,8,11,63,64,65,66]. In addition, clinical trials for the concomitant therapy of calcitriol and other antitumor agents revealed the potential of calcitriol as an accompanying agent. As a possible clinical setting, we assumed the intravenous administration of 74 μg calcitriol, which is the suggested MTD when calcitriol is used alone [39]. If the MTD of calcitriol is changed when used in combination with other agents, the exposure to calcitriol may be altered, leading to different effects on the substrate drugs of CYPs. Calcitriol has also been evaluated for its potential use in other conditions such as inflammation, thrombosis, and coronavirus disease 2019 (COVID-19) using various dose regimens [67,68,69]. Therefore, if the calcitriol dose is higher than that assumed in this study or the systemic exposure is more than that applied here, the clinical significance of CYP induction by calcitriol may need to be reinvestigated using the new pharmacokinetic data.

It has been reported that cryopreserved hepatocytes present a similar pattern of CYP induction as freshly isolated hepatocytes, making the former a valuable tool to study the induction of CYPs [70,71,72,73]. However, there are obvious limitations to using cryopreserved human hepatocytes. The variability in the results for metabolizing enzymes is substantial depending on donors as well as experimental conditions [52,74]. In some cases, the limited number of hepatocytes that can be obtained from one donor may be an obstacle for repeated tests or study extensions. In contrast, HepaRG cells can offer significant advantages over hepatocytes in terms of data variability, costs, and ease of handling [34]. It has also been reported that the genes involved in drug metabolism in HepaRG cells are regulated in a similar manner as those in human hepatocytes [33], these cells have been widely used as a tool for the study of drug metabolizing enzymes. In this study, the induction profiles of CYP3A4 in HepaRG were similar to those in hepatocytes from donor #3, which showed the highest mRNA fold-induction among the three lots of hepatocytes. Considering that CYP3A is responsible for the metabolism of more than 50% of all the drugs that are CYP substrates [75] and given that a conservative approach should be taken when estimating the potential of DDIs in vitro, we can assume that HepaRG cells are a suitable tool to estimate the clinical significance of CYP3A4 induction.

Calcitriol is known to regulate various drug-metabolizing enzymes by binding with the vitamin D receptor (VDR). These metabolizing enzymes include CYP2B6, 3A4, 2C8, and 2C9 [30,50,76]. In the present study, calcitriol induced CYP2B6, 3A4, 2C8, and 2C9 in both hepatocytes and HepaRG cells, and this induction may be VDR-dependent, as reported previously. In the case of CYP2C19, its mRNA expression was upregulated in the presence of 100 nM calcitriol in HepaRG cells only, and a similar increase was not observed in hepatocytes. To the best of our knowledge, it is known that only the pregnane X receptor (PXR) and constitutive androstane receptor (CAR) are involved in the regulation of CYP2C19 and calcitriol is not associated with these transcription factors [77]. The mechanism of CYP2C19 upregulation by calcitriol in HepaRG cells and the reason for the difference in CYP2C19 induction profiles between hepatocytes and HepaRG need to be investigated further to understand the effect of calcitriol on CYP induction more clearly. Nevertheless, CYP induction by calcitriol treatment is not expected to be clinically significant even for CYP2C19 because its mRNA increased only three-fold in the presence of 100 nM calcitriol, which is a much higher concentration than that observed with the dosage regimens used in this study.

## 5. Conclusions

This is the first report to predict clinical implications of DDIs via CYP induction by calcitriol. Calcitriol upregulated several CYP isozymes in vitro; however, it was predicted that calcitriol does not affect the pharmacokinetic properties of substrate drugs of the CYP isozymes in several clinical settings using various pharmacokinetic estimation models. Therefore, we conclude that CYP induction by calcitriol treatment would not be clinically significant under typical clinical conditions.

## Figures and Tables

**Table 1:** Information about each parameter used for the calculation of the area under the plasma concentration-time curve ratio (*AUCR*).

Parameters	Definition	Equation or value used	Source
C_g_	Effect of induction in gut	$1+\frac{d\times E_{max}\times {\left[I\right]}_{g}}{{\left[I\right]}_{g}+EC_{50}}$	[32]
C_h_	Effect of induction in liver	$1+\frac{d\times E_{max}\times {\left[I\right]}_{h}}{{\left[I\right]}_{h}+EC_{50}}$	[32]
F_g_	Fraction available after intestinal metabolism	1	[32]
f_m_	Fraction of hepatic clearance of the substrate mediated by the cytochrome P450 (CYP) enzyme that is subject to induction	1	
d	Scaling factor	1	[32]
[I]_g_	Concentration of calcitriol in gut	$F_{a}\times K_{a}\times \frac{Dose}{Q_{en}}$	[40]
[I]_h_	Concentration of calcitriol in liver	$f_{u,p}\times (C_{max}+(\frac{F_{a}\times F_{g}\times K_{a}\times Dose}{Q_{h}\times RB}))$	[41]
f_u,p_	Unbound fraction in plasma	0.01	[32]
F_a_	Fraction absorbed after oral administration	1	[42]
K_a_	First order absorption rate constant in vivo	0.1 min^−1^	[41]
Q_h_	Hepatic blood flow	97 L/h/70 kg	[42]
Q_en_	Blood flow through enterocytes	18 L/h/70 kg	[43]
RB	Blood-to-plasma concentration ratio	1	

**Table 2:** Maximum induction effect (*E_max_*) and half maximal effective concentration (*EC_50_*) values for CYP mRNA induction by calcitriol treatment.

CYPs	Parameter	Hepatocyte #1	Hepatocyte #2	Hepatocyte #3	HepaRG
CYP2B6	E_max_	NA ^a^	NA	NA	9.647
	EC_50_ (nM)	NA	NA	NA	45.330
CYP3A4	E_max_	50.120	4.462	11.560	39.650
	EC_50_ (nM)	49.110	44.270	32.560	25.850
CYP2C8	E_max_	2.879	2.305	NA	6.941
	EC_50_ (nM)	2.733	2.239	NA	71.850
CYP2C9	E_max_	2.141	NA	NA	4.208
	EC_50_ (nM)	2.461	NA	NA	32.110

^a^ NA: Not available.

**Table 3:** R_3_ values calculated using the basic kinetic model.

CYPs	Dose Regimen	Hepatocyte #1	Hepatocyte #2	Hepatocyte #3	HepaRG
CYP2B6	Calcitriol standard PO regimen ^a^	NA ^d^	NA	NA	0.886
	Calcitriol standard IV regimen ^b^	NA	NA	NA	***0.605***
	Calcitriol high-dose IV regimen ^c^	NA	NA	NA	***0.168***
CYP3A4	Calcitriol standard PO regimen	***0.618***	0.943	0.824	***0.522***
	Calcitriol standard IV regimen	***0.282***	***0.800***	***0.537***	***0.183***
	Calcitriol high-dose IV regimen	***0.039***	***0.301***	***0.127***	***0.037***
CYP2C8	Calcitriol standard PO regimen	***0.655***	***0.669***	NA	0.945
	Calcitriol standard IV regimen	***0.415***	***0.446***	NA	***0.767***
	Calcitriol high-dose IV regimen	***0.269***	***0.312***	NA	***0.264***
CYP2C9	Calcitriol standard PO regimen	***0.701***	NA	NA	0.927
	Calcitriol standard IV regimen	***0.475***	NA	NA	***0.719***
	Calcitriol high-dose IV regimen	***0.329***	NA	NA	***0.284***

PO: per os (oral), IV: intravenous. ^a^ Calcitriol standard PO regimen: steady-state maximum plasma concentration (C_max_) after intake of 1 μg calcitriol (assumed maximum oral dose for the approved indication) was used [37]. ^b^ Calcitriol standard IV regimen: C_max_ after intravenous administration of 4 μg calcitriol (assumed maximum IV dose for the approved indication) was used [38]. ^c^ Calcitriol high-dose IV regimen: C_max_ after intravenous administration of 74 μg calcitriol (the reported maximum tolerable dose (MTD) of IV calcitriol from the clinical study) was used [39]. ^d^ NA: not available. Italic bold font indicates that the calculated R_3_ value is below or equal to the predefined cut-off value (0.8).

**Table 4:** Calculated AUCR values using static mechanistic model.

CYPs	Dose Regimen	Hepatocyte #1	Hepatocyte #2	Hepatocyte #3	HepaRG
CYP2B6	Calcitriol standard PO regimen ^a^	NA ^d^	NA	NA	1.000
	Calcitriol standard IV regimen ^b^	NA	NA	NA	0.998
	Calcitriol high-dose IV regimen ^c^	NA	NA	NA	0.967
CYP3A4	Calcitriol standard PO regimen	0.998	1.000	0.999	0.997
	Calcitriol standard IV regimen	0.989	0.999	0.996	0.983
	Calcitriol high-dose IV regimen	0.858	0.984	0.946	0.802
CYP2C8	Calcitriol standard PO regimen	0.998	0.998	NA	1.000
	Calcitriol standard IV regimen	0.989	0.989	NA	0.999
	Calcitriol high dose IV regimen	0.861	0.865	NA	0.985
CYP2C9	Calcitriol standard PO regimen	0.998	NA	NA	1.000
	Calcitriol standard IV regimen	0.991	NA	NA	0.999
	Calcitriol high-dose IV regimen	0.883	NA	NA	0.979

PO: per os (oral), IV: intravenous. ^a^ Calcitriol standard PO regimen: steady-state C_max_ after intake of 1 μg calcitriol (assumed maximum oral dose for the approved indication) was used [37]. ^b^ Calcitriol standard IV regimen: C_max_ after intravenous administration of 4 μg calcitriol (assumed maximum IV dose for the approved indication) was used [38]. ^c^ Calcitriol high-dose IV regimen: C_max_ after intravenous administration of 74 μg calcitriol (the reported MTD of IV calcitriol) was used [39]. ^d^ NA: Not available.

**Table 5:** Predicted pharmacokinetic parameters for midazolam with or without calcitriol treatment.

Data Source	Geometric Mean (90% Confidence Interval)				Geometric Mean Ratio (Midazolam+Calcitriol /Midazolam)	
	Midazolam		Midazolam + Calcitriol			
	C_max_ (ng/mL)	AUC_0–24h_^a^ (ng/mL·h)	C_max_ (ng/mL)	AUC_0–24h_ (ng/mL·h)	C_max_	AUC_0-24h_
Hepatocytes #1	12.18 (11.25, 13.17)	35.50 (32.54, 38.74)	12.16 (11.24, 13.16)	35.39 (32.44, 38.61)	0.9989	0.9969
Hepatocytes #2			12.17 (11.25, 13.17)	35.48 (32.52, 38.72)	0.9998	0.9994
Hepatocytes #3			12.17 (11.25, 13.17)	35.46 (32.50, 38.69)	0.9995	0.9988
HepaRG			12.16 (11.23, 13.15)	35.35 (32.40, 38.56)	0.9985	0.9955

^a ^AUC_0–24h_: area under the concentration–time curve from 0 to 24 h.

## Data Availability

The data presented in this study are available in the article and Supplementary Materials.

## Supplementary Materials

The following are available online at https://www.mdpi.com/1999-4923/13/2/181/s1, Figure S1: Input parameters used for PBPK model development of calcitriol, Table S1: Observed and simulated plasma concentration–time profiles after intravenous administration of 74 μg calcitriol.
