# Supplementary material for: Pharmacokinetic Estimation Models-based Approach to Predict Clinical Implications for CYP Induction by Calcitriol in Human Cryopreserved Hepatocytes and HepaRG Cells
Source: Pharmaceutics. 2021 Jan 29;13(2):181. doi: 10.3390/pharmaceutics13020181 (PMC7911399; doi:10.3390/pharmaceutics13020181)
Supplement: Supplementary file 1 [file pharmaceutics-13-00181-s001.pdf]

pharmaceutics

Supplementary Materials: Pharmacokinetic Estimation Models-based Approach to Predict Clinical Implications for CYP Induction by Calcitriol in Human Cryopreserved Hepatocytes and HepaRG Cells

Yoon-Jee Chae, Min-Soo Kim, Suk-Jae Chung, Mi-Kyung Lee, Kyeong-Ryoon Lee* and Han-Joo Maeng*

Table S1. Input parameters used for physiologically-based pharmacokinetic (PBPK) model development of calcitriol.

Parameter	Value	Reference
	**Physicochemical properties**	
Molecular weight (g/mol)	416.637	
LogP	5	
Compound type	Neutral	
Blood-to-plasma partition ratio	1	
Hematocrit	45	Default
Fraction unbound in plasma	0.01	[35]
	Distribution (Full PBPK model)	
K_p scalar	0.005	Parameter estimation
V_{ss} (L/kg)	0.348	Calculated using K_p scalar and Rodgers and Rowland methods
$K_{p,\ liver}$	0.212	Calculated using K_p scalar and Rodgers and Rowland methods
	Elimination (in vivo clearance)	
CL (L/h)	1.675	[37]

K_p scalar: scaling factor used to scale the tissue-to-plasma partitioning coefficient; V_{ss}: volume of distribution at steady-state; $K_{p,\ liver}$: liver-plasma partition coefficient; CL: clearance.

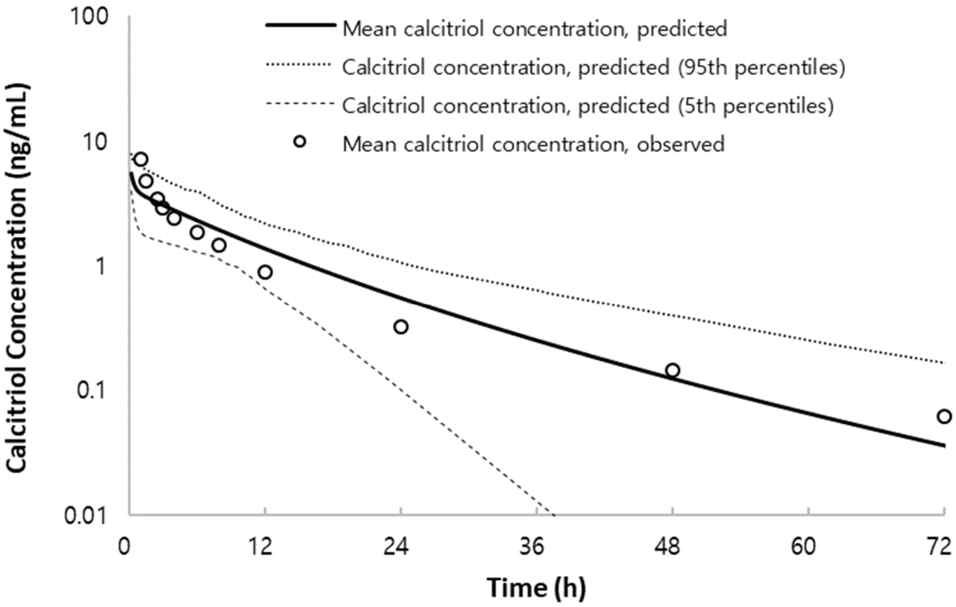

Figure S1. Observed and simulated plasma concentration–time profiles after intravenous administration of 74 μg calcitriol. The black line represents simulated mean calcitriol concentration and empty circles represent observed mean calcitriol concentrations.
